# Role Medium-Chain Fatty Acids in the Lipid Metabolism of Infants

**DOI:** 10.3389/fnut.2022.804880

**Published:** 2022-06-09

**Authors:** Tinglan Yuan, Lei Wang, Jun Jin, Lijuan Mi, Jinzhu Pang, Zhengdong Liu, Jinyan Gong, Cong Sun, Jufang Li, Wei Wei, Qingzhe Jin, Xingguo Wang

**Affiliations:** ^1^Collaborative Innovation Centre of Food Safety and Quality Control in Jiangsu Province, School of Food Science and Technology, Jiangnan University, Wuxi, China; ^2^Inner Mongolia Mengniu Dairy (Group) Co., Ltd., Beijing, China; ^3^Yashili International Group Co., Ltd., Guangzhou, China; ^4^Zhejiang Provincial Key Lab for Biological and Chemical Processing Technologies of Farm Product, School of Biological and Chemical Engineering, Zhejiang University of Science and Technology, Hangzhou, China; ^5^College of Food Science and Technology, Henan University of Technology, Zhengzhou, China

**Keywords:** medium-chain fatty acids, human milk fat, infant formula, lipid metabolism, medium-chain triacylglycerols

## Abstract

Human breastmilk, the ideal food for healthy infants, naturally contains a high concentration of medium-chain fatty acids (MCFAs, about 15% of total fatty acids). MCFAs are an important energy source for infants due to their unique digestive and metabolic properties. MCFA-enriched oils are widely used in an infant formula, especially the formula produced for preterm infants. Recently, there has been a growing interest in the triglyceride structure of MCFAs in human milk, their metabolism, and their effects on infant health. This study summarized the MCFA composition and structure in both human milk and infant formula. Recent studies on the nutritional effects of MCFAs on infant gut microbiota have been reviewed. Special attention was given to the MCFAs digestion and metabolism in the infants. This paper aims to provide insights into the optimization of formulations to fulfill infant nutritional requirements.

## Introduction

Dietary nutrition is vital for the metabolic outcome and development of infants. Human milk is the optimal source of nutrition for infants. Fat is an important component of milk, supplying ∼50% of energy for infants (1). Human milk fat contains approximately 50 kinds of fatty acids (FAs), mostly in the form of triacylglycerols (TAGs), which are more than 98% of the fat (2). Human milk is a natural source of medium-chain fatty acids (MCFAs), comprising approximately 10–35% of the total FAs (3), and half of the TAG molecules in human milk contain MCFAs (4). MCFAs have great importance for infants with an immature digestive system (5).

By definition, MCFAs generally refer to saturated FAs, with a chain length of 6–12 carbons, naturally occurring in some vegetable oils (coconut and palm kernel oils) and milk fat (5, 6). Typical medium-chain TAG (MCT) is mainly composed of caprylic acid (8:0) and capric acid (10:0). In milk fat, MCFAs are generally regarded as saturated FAs, with a chain length of 8–14 (7–10). During lactation, the mammary epithelial cells are the primary site for *de novo* FA synthesis, and the presence of an acyl thioester-hydrolase limits FA synthesis to more than 16 (11, 12); therefore, the main FAs synthesized *de novo* in mammary glands, such as 8:0,10:0, 12:0, and 14:0, are known as MCFAs on the basis of the source of milk FAs, which was adopted in this review.

MCFA-enriched oils, such as coconut oil and palm kernel oil or MCT, are often added to infant formulas as a source of MCFAs to facilitate fat absorption and the growth of infants, especially in formulas designed for low birth weight or preterm infants (13, 14). MCFAs are an important source of energy because their properties during the processes of digestion, absorption, and metabolism are different from those of long-chain fatty acids (LCFAs) (6, 15). It has been well documented that MCFAs are more easily absorbed and oxidized for energy than LCFAs since they are primarily absorbed directly to the liver *via* the portal vein and rapidly transferred independently of the carnitine shuttle system to the mitochondria (6). The addition of MCT to infant formula could facilitate better absorption of lipids (16). MCFAs also display antiviral and antibacterial activities and have a functional impact on the modulation of the gut microbiota during early infancy (17, 18), which is related to the maturation of the immune system and general health (19).

As an important energy source, the role of MCFAs in lipid metabolism in infants deserves more attention. However, a recent updated systematic review has concluded that an MCT formula did not improve preterm infant growth or have fewer adverse effects (20). Knowledge of human milk and infant formulas has greatly increased, which will help to understand better the health benefits of different diets. In this paper, we focus on the advances and controversies in the MCFA nutrition of infants. This work is intended to provide a holistic review of MCFAs, from structural characteristics to metabolic effects in infants.

## Medium-Chain Fatty Acids in Human Milk and Infant Formulas

Human milk fat was considered the reference standard of an infant formula. The MCFAs in human milk have been well studied as important saturated fatty acids, and their content was influenced by multiple factors (21). The MCFAs in an infant formula depend on the oil species supplied. The difference in MCFA between human milk and the infant formula was seldom compared. In this study, MCFA content in human milk fat and the infant formula fat were compared with emphasis on the differences between TAG structures.

### Medium-Chain Fatty Acids Composition of Human Milk Fat

Medium-chain fatty acids (8:0–14:0) account for 7–23% of the total FAs in human milk, and 12:0 and 14:0 are predominant, accounting for approximately 5% and 6%, followed by 10:0 and 8:0 (Table 1). It was found that the MCFA composition of human milk changes throughout lactations, with a lower amount in colostrum than in transitional and mature milk (22–27), which may be attributed to the immature metabolism of the mammary gland or the biosynthetic capacity in early lactation (12). A pooled data analysis suggested that MCFAs are comparable between preterm and term milk (26). Besides physiological factors, maternal dietary, and sociodemographic and environmental factors are associated with the MCFA composition of human milk (21). It has been suggested that rich n-3 LCFAs or high-carbohydrate and low-fat diet could stimulate the synthesis of *de novo* FAs in the cytoplasm of the mammary glands (1, 11), resulting in a higher content of MCFAs in human milk (9, 28, 29). Actually, lower levels of MCFAs were found in the human milk of obese mothers (BMI of over 30) relative to the overweight group (BMI between 25 and 30), which may have been caused by the high fat intake (30). Additionally, when mothers or infants suffered cold-like symptoms, the proportions of 10:0 and 12:0 of FAs in human milk were significantly lower (10).

**TABLE 1:** Content of MCFAs and TAGs containing MCFAs in human milk*.

Country/area	Sample information	MCFAs 8:0-14:0	TAGs containing 8:0-14:0	Ref.
Wuxi, China	Pretermmature milk, *n* = 30	9.30	29.16	(4)
	Full-termmature milk, *n* = 30	8.70	29.88	
Zhengzhou, China	Mature milk, *n* = 30	11.26	40.09	(36)
Wuhan, China	Mature milk, *n* = 30	8.51	31.32	
Harbin, China	Mature milk, *n* = 30	7.54	31.77	
Wuxi, China	Colostrum, *n* = 103	8.04	22.57	(22, 32)
	Transitional milk, *n* = 103	13.65	34.73	
	Mature milk, *n* = 103	12.10	31.90	
Beijing, China	Colostrum, *n* = 126	7.43	16.95	(24)
	Mature milk, *n* = 40	10.32	22.54	
Hubei, China	Transitional milk, *n* = 9	–	27.10	(33)
Sichuan, China	Transitional milk, *n* = 8	–	31.53	
Beijing, China	Transitional milk, *n* = 10	–	28.29	
Hubei, China	Mature milk, *n* = 9	–	47.11	
Sichuan, China	Mature milk, *n* = 8	–	42.26	
Beijing, China	Mature milk, *n* = 10	–	38.56	
Beijing, China	Mature milk, *n* = 10	8.00^a^	31.51^b^	(31)
Finland	Mature milk, *n* = 10	13.27^a^	40.54^b^	
Denmark	Colostrum, *n* = 45	10.68	31.15	(35)
	Transitional milk, *n* = 45	18.17	46.20	
	Mature milk, *n* = 45	23.12	40.35	
Italy	Mature milk, *n* = 1	18.57^a^	30.80	(34)

*Data on human milk for infants < 12 months of age; the values were estimated on the basis of the mean weight percentage (wt%) of total TAGs, unless otherwise indicated. “–” indicated the information was not reported. ^a^ Values were mole%. ^b^ The TAGs less than.1% were not included. TAGs, triacylglycerols; MCFAs, medium-chain fatty acids.

### Triacylglycerol Containing Medium-Chain Fatty Acids Composition of Human Milk Fat

Medium-chain fatty acids account for a small portion of human milk; however, recent studies have shown that approximately half of the TAG molecule species in human milk contain at least one MCFA (31, 32). Table 1 shows the content of the TAGs, containing MCFAs (8:0-14:0) in human milk collected in four countries, summarized from eight publications (4, 24, 31–36). The content varies from 16.95% to 47.11% of total TAG. Among these TAGs, only a few TAGs composed of three MCFAs (0.80–1.13%.) were detected. MCFAs are naturally present in human milk as medium- and long-chain triacylglycerols (MLCTs). Particularly, it was recognized that the MCFAs mainly existed together with 16:0 and 18 FAs (18:0, 18:1 or 18:2), such as 12:0/16:0/18:1 (LaPO), 12:0/18:1/18:2 (LaOL), and 12:0/18:1/18:1 (LaOO) (37).

### Comparison of Medium-Chain Fatty Acids in Human Milk Fat and Infant Formula Fat

Human milk fat is well-known as the best nutrition for infants and is generally considered the reference standard for the development of an infant formula. Our group has recently analyzed the FA and TAG composition of 180 commercial infant formulas on the Chinese market (38, 39). As for MCFAs, we have found that almost all of the formulas contained higher amounts of 8:0 than mature human milk. Infant formulas supplied with coconut oil generally contained more 12:0 than human milk (38, 40).

The main TAGs containing MCFAs were 12:0/12:0/12:0 (LaLaLa) and 12:0/12:0/14:0 (LaLaM) in the plant oil-based infant formulas. Short- and medium-chain TAGs, such as 4:0/12:0/16:0 (BuLaP), 4:0/14:0/16:0 (BuMP), and 6:0/14:0/16:0 (CoMP), were predominant in the cow milk or goat milk-based infant formulas (39). These compounds are very different from those naturally present in human milk (37). The significant differences in TAG molecular species containing MCFAs between human milk and infant formulas should be given more attention in the future, as they could lead to altered lipid metabolism and physiological health status.

## Roles of Medium-Chain Fatty Acids in Lipid Digestion and Absorption

### Gastric Digestion and Absorption

The digestion of lipids begins in the stomach, where the FAs are released by gastric lipase, which is present from the 11th week of gestation and reaches adult activity levels at birth (41). It has been shown that the gastric hydrolysis of fat is higher in infants fed with human milk than those fed with a formula, including the MCT formula, although the 8:0 and 10:0 were less abundant in human milk (approximately 2%) than those in an infant formula (approximately 24%) (42, 43). Moreover, a crossover study showed that MCFAs in the MCT formula (42% MCT) and the LCT formula (7% MCT) fed to 12 preterm infants were hydrolyzed to the same extent (44), suggesting that MCT supplementation has no improvement on gastric lipolysis, possibly because of the stimulation of lipase secretion by LCFAs (44, 45). Still, short-chain FAs and MCFAs could be absorbed directly through the stomach wall and enter the portal vein, so even MCT molecules could be partially absorbed (44, 46), which provides infants with a readily available energy source.

### Intestinal Digestion and Absorption

**FIGURE 1:**
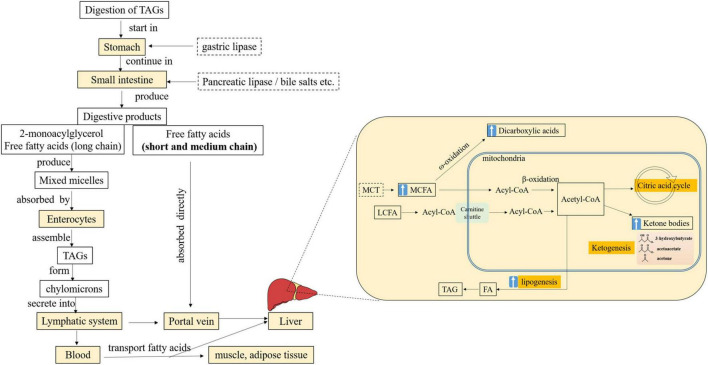
Simplified schematic of digestion, absorption, and transport of MCFAs from TAGs and the metabolism of MCFAs in the liver. FA, fatty acid; MCFA, medium-chain fatty acid; MCT, medium-chain triacylglycerol; LCFA, long-chain fatty acid; TAG, triacylglycerol; “↑,” enhanced. Adopted from (48).

Ingested fat is digested and absorbed primarily in the small intestine, where TAGs are mainly hydrolyzed by pancreatic lipase to form the primary products 2-monoacylglycerol and free FAs (47). Subsequently, these products and bile salts form mixed micelles and reach the enterocytes, where they are absorbed, resynthesized as TAGs, and packaged as chylomicrons. Alternatively, short-chain FAs and MCFAs can leave the intestine and are directly and rapidly released into the portal circulation (Figure 1; 48).

With MCFAs being absorbed easier and faster than LCFAs, it is expected that adding MCTs to formulas would promote the higher absorption of fat (49). Some studies have significantly improved fat absorption by infants fed the MCT formula compared with the LCT formula (50–52). However, other studies have indicated that the fat absorption in infants was similar irrespective of the MCT levels (45, 53). Additionally, a lower fat absorption from formulas than human milk was reported to be common (54), which may be partly affected by the different structures of TAGs containing MCFAs (MLCT vs. MCT/LCT). Previous animal studies have demonstrated that structured lipids MLCT from esterification of MCT and fish oil had a higher lymphatic absorption than the mixture of MCT/LCT (55, 56). The differences in hydrolysis products and FAs released from different TAGs may influence the production of some hormones that control the secretion of pancreatic enzymes and lipid digestion, causing different digestive activities (57). Still, the exact mechanism is not fully understood.

## Modulation of the Gut Microbiota and Intestinal Development

MCFAs as the intestinal energy sources can also improve the growth performance of infants by improving intestinal function. The protective effect of MCFAs and MCT on the intestinal barrier and gut health has been supported in suckling piglets as an *in vivo* mammalian neonate model (58, 59). Studies have demonstrated that MCFAs in milk, especially 8:0, 10:0, and 12:0, have antimicrobial effects against several bacteria, such as *Clostridium*, *Salmonella*, and *Helicobacter pylori*, which might enhance resistance against intestinal pathogens (17, 60, 61). A recent study has determined that dietary supplementation with MCTs reduced the colonization of *Candida* in preterm infants (62). Nevertheless, there are no reports of how the MLCT structure in human milk being different from an infant formula would impact the establishment of the microbiota or gut-associated function in infants.

## Lipid Metabolism of Medium-Chain Fatty Acids in Infants

### Oxidation Metabolism

The metabolic fate of MCFAs is chiefly catabolism by the liver, where the major pathway for FAs is β-oxidation in mitochondria. LCFAs cross the mitochondrial membrane with the aid of carnitine, whereas MCFAs can enter independent of the carnitine transport system and undergo preferential oxidation by the tricarboxylic acid cycle (6), and the oxidation is not subject to inhibition by malonyl CoA (Figure 1; 48). Thus, the rapid and almost complete oxidation of MCT has been suggested.

Nevertheless, there might be a limit to the amount of MCT being completely oxidized by preterm infants. For example, in infants fed a formula containing 40% fat as MCT, an average of no more than 47% of the administered 8:0 was oxidized (63). Furthermore, Whyte et al. (64) found no significant differences in the rate of energy expenditure and energy storage between the MCT formula and the LCT formula, which meant that MCT was not oxidized, and portions undergo the same metabolic fate as LCT. Additionally, infants fed a commercial formula might store up to 12% of the MCFAs (8:0–10:0) in subcutaneous fat (65). Hence, incorporating high-level MCT into a formula will not necessarily improve the neonate’s ability to consume or metabolize energy (66).

Another consequence of MCFAs (MLCT or MCT) being easily oxidized is the effects on body fat accumulation and obesity, which have been well demonstrated in animal and clinical trials (67–69). MCFAs may enhance mitochondrial function, lipid oxidation, and thermogenesis by modulating cellular signaling and regulating key circulating metabolites and hormones (70). In a rodent model, consumption of MCFAs in early life has been shown to prevent excessive fat accumulation and insulin sensitivity in adulthood (71). While limited in infants, the evidence points to a positive impact of MCFAs on obesity.

Acetyl CoA can also be converted into ketone bodies, such as acetoacetate, 3-hydroxybutyrate (β-HB), and acetone, in the liver mitochondria (Figure1). Extrahepatic tissues, including the brain, can use ketone bodies as fuel. For newborns and older infants, ketones are an essential and important source of energy for the brain (72). MCFAs, in particular, are ketogenic and the ideal ketone precursors. A higher level of plasma β-HB was observed in the preterm infants fed with the MCT formula compared with a control formula without MCT (53). However, a significantly higher level of ketone bodies (β-HB and acetoacetate) was seen in breastfed infants than in formula-fed infants, although the content of MCFAs in infant formula was not reported (73). The relationship between TAG structure (MLCT or MCT) and the level of ketone bodies remains to be further studied.

### Lipogenesis Metabolism

Besides an increased metabolic rate, MCFAs are associated with increased lipogenesis in the liver (74, 75). MCTs may cause accelerated MCFA oxidation and promote the production of acetyl CoA, which can be a substrate providing carbons for chain elongation and FA synthesis (Figure 1). In preterm infants fed the MCT formula (38%), greater lipogenesis was observed, which might partly explain the incomplete oxidation (76). Increased lipogenesis may increase hepatic fat accumulation and metabolic burden, as well as interfere with the metabolism of other FAs in infants. It was observed that the levels of plasma phospholipid 22:6 n-3 were significantly lower in preterm infants fed the high MCT (46% of 8:0 and 10:0) formula than that low MCT formula (4.8% of 8:0 and 10:0, similar content of 18:2 and 18:3) (77). Recently, in 47 clinically stable preterm infants, Billeaud et al. (78) have observed that human milk supplemented with a fortifier containing MCFAs (8:0 and 10:0, 12.5%) increased the levels of plasma n-9 monounsaturated FA significantly.

Although the impact of MLCT on infant health remains unclear, numerous clinical studies have demonstrated that, compared with a physical mixture of MCT and LCT, MLCT exerted some favorable effects on nutrition status in surgical patients, in particular lipid metabolism and liver function (79, 80). Hence, we can speculate that the MLCT in human milk may be more beneficial for lipid metabolism and development in infants, which must be verified in future studies.

## Conclusion and Future Perspectives

Milk fat is naturally rich in MCFAs, which are very important to the growth and development of infants. Although the consensus is that MCFAs have a unique advantage in absorption and metabolism in infants, the benefits of MCT on the growth performance of infants are not clearly shown in clinical trials. It is necessary to determine how the TAG structure of MCFAs influences lipid digestion and absorption and causes the observed outcome. Moreover, the effects of different MCFAs (e.g., 12:0 and 14:0) on lipid metabolism warrant further investigation. Additionally, the distinct metabolic effects on infants resulting from the differences in the composition of TAGs containing MCFAs between an infant formula and human milk have not been fully addressed. The molecular species of TAGs containing MCFAs in natural fats, their metabolic processing, and the potential health benefits for infants are of great interest. We present a summary of recent studies on MCFA composition in human milk and an appraisal of the function and roles of MCFAs on long-term metabolism in infants, which will be conducive to the development of infant formulas.

## Author Contributions

WW and JL designed the review. TY wrote the original manuscript. WW, LW, JJ, LM, JP, ZL, JG, CS, QJ, and XW reviewed and edited the manuscript. All authors read, discussed, and agreed to the published version of the manuscript.

## Conflict of Interest

LM, JP, and JL were employed by Inner Mongolia Mengniu Dairy (Group) Co., Ltd. ZL was employed by Yashili International Group Co., Ltd. The remaining authors declare that the research was conducted in the absence of any commercial or financial relationships that could be construed as a potential conflict of interest.

## Publisher’s Note

All claims expressed in this article are solely those of the authors and do not necessarily represent those of their affiliated organizations, or those of the publisher, the editors and the reviewers. Any product that may be evaluated in this article, or claim that may be made by its manufacturer, is not guaranteed or endorsed by the publisher.

## Abbreviations

FA: fatty acids
LCFAs: long-chain fatty acids
MCFAs: medium-chain fatty acids
MCT: medium-chain triacylglycerol
MLCTs: medium- and long-chain triacylglycerols
TAGs: triacylglycerols.
